# In Vitro Cellular Uptake Studies of Self-Assembled Fluorinated Nanoparticles Labelled with Antibodies

**DOI:** 10.3390/nano11081906

**Published:** 2021-07-24

**Authors:** Mona Atabakhshi-Kashi, Mónica Carril, Hossein Mahdavi, Wolfgang J. Parak, Carolina Carrillo-Carrion, Khosro Khajeh

**Affiliations:** 1 Department of Nanobiotechnology, Tarbiat Modares University, Tehran 14115-175, Iran; monaatabakhshi@gmail.com; 2 Bioengineered Particles Group, CIC biomaGUNE, 20014 San Sebastian, Spain; monica.carrilg@ehu.eus (M.C.); wolfgang.parak@uni-hamburg.de (W.J.P.); 3 Ikerbasque, Basque Foundation for Science, 48013 Bilbao, Spain; 4 Instituto Biofisika UPV/EHU, CSIC, 48940 Leioa, Spain; 5 Departamento de Bioquímica y Biología Molecular, UPV/EHU, 48940 Leioa, Spain; 6 School of Chemistry, College of Science, University of Tehran, Tehran 14174-66191, Iran; hosinmahdavi@ut.ac.ir; 7 Fachbereich Physik and CHyN, Universität Hamburg, 22607 Hamburg, Germany; 8 Institute for Chemical Research (IIQ), CSIC-University of Seville, 41092 Sevilla, Spain

**Keywords:** nanoparticles, quantum dots, antibody conjugation, fluorination, self-assembly

## Abstract

Nanoparticles (NPs) functionalized with antibodies (Abs) on their surface are used in a wide range of bioapplications. Whereas the attachment of antibodies to single NPs to trigger the internalization in cells via receptor-mediated endocytosis has been widely studied, the conjugation of antibodies to larger NP assemblies has been much less explored. Taking into account that NP assemblies may be advantageous for some specific applications, the possibility of incorporating targeting ligands is quite important. Herein, we performed the effective conjugation of antibodies onto a fluorescent NP assembly, which consisted of fluorinated Quantum Dots (QD) self-assembled through fluorine–fluorine hydrophobic interactions. Cellular uptake studies by confocal microscopy and flow cytometry revealed that the NP assembly underwent the same uptake procedure as individual NPs; that is, the antibodies retained their targeting ability once attached to the nanoassembly, and the NP assembly preserved its intrinsic properties (i.e., fluorescence in the case of QD nanoassembly).

## 1. Introduction

Continuous advances in the engineering of nanoparticles (NPs) are giving rise to a myriad of nanostructured systems for their use in a wide range of potential biological and biomedical applications [1,2], extending from cell tracking (for diagnostic use) [3,4] to drug delivery (for therapeutic purposes) [5,6]. Tailored NP properties can be achieved through surface functionalization strategies, where a plethora of different ligands (i.e., functional groups) may be used to modulate their biological behavior (i.e., nano-bio interactions) with a two-fold aim: (i) to increase the NPs’ accumulation at sites of interest, and decrease non-specific uptake in other areas of the body (i.e., selective targeting capabilities); and (ii) to increase the rate of NPs’ internalization for improving the sensitivity/efficacy of diagnostic/therapeutic functions, respectively [7,8,9,10,11]. One of the most common modifications involves decorating the NPs’ surface with multiple affinity ligands (multivalency) in order to actively target nanoparticles to specific pathology-associated biomarkers, as, for example, overexpressed cell membrane receptors in cancer cells [8,9,12].

In this context, the conjugation of monoclonal antibodies (mAbs) to NPs is widely used to trigger the internalization in cancer cells via receptor-mediated endocytosis [13,14]. However, the chemistry behind the antibody conjugation can be very challenging for several reasons. First, the conjugation method used to immobilize the antibody on the NPs’ surface has to be properly selected in order to avoid potential undesired effects such as nanoparticle aggregation and complete (or partial) antibody denaturation, which would lead to poor target recognition of the surface-bound antibodies. In this regard, several conjugation methods have been explored in search of a general immobilization protocol [15,16,17]. Second, the final performance of the antibody-conjugated NPs also depends significantly on the antibody orientation [15,17,18,19,20], the number of immobilized antibodies per NP or ligand density (antibody surface coverage) [15,18,21], the number of accessible Fab and Fc domains [17,22], as well as the potential interactions with neighboring ligands when working with dual-functionalized NPs [23]. Therefore, a careful optimization of all of these aspects is a must in view of a future translation of these nanosystems to the clinical practice. It is also well known that in vivo targeting imposes additional hurdles [12], and thus this report here entirely focuses on in vitro uptake only.

Despite the already demonstrated capabilities of antibody-conjugated NPs for targeting cells [24,25], ligand-mediated uptake is not always as clear as expected [26]. The formation of a protein corona is one of the reasons for reduced targeting efficiency of Ab-labeled NPs [27]. Numerous efforts have been directed towards understanding the mechanisms and parameters involved in the cellular uptake of NPs [7,28,29,30]. For example, it is well known that the NP size plays an important role in cellular uptake [11,31,32,33]. Apart from biological effects, when using in vitro cell culture exposure models, stronger sedimentation for bigger NPs plays a role in their enhanced uptake [34]. In the particular case of antibody-conjugated NPs, around 40–50 nm has been reported to be a good size for NP uptake due to a proper balance of the two processes involved in receptor-mediated endocytosis: multivalent crosslinking of membrane receptors, and membrane wrapping [31]. Theoretical models have demonstrated that NP nanoassemblies have higher rates of internalization than single NPs [35]. In this regard, the formation of ca. 50 nm-assemblies when working with very small NPs (<10 nm) may be advantageous. By coupling several small NPs such as quantum dots (QDs) in a matrix to give bigger assemblies (around 50 nm in diameter), uptake behavior would be improved while maintaining the optical properties of the QDs. However, for such bigger NP assemblies, their size is much larger than the size of Abs, whereas, in the case of individual QDs, their size is comparable to that of Abs, which could affect the effect of the Ab-receptor binding. 

Several works have already reported the preparation of QD nanoassemblies, either using QDs alone [36], combining QDs with other types of NPs such as Au NPs [37], Fe_3_O_4_ magnetic NPs [38], or silica NPs [39], to finally construct functional platforms for cellular labeling or intracellular delivery of a payload with fluorescence monitoring. For example, Rotello et al. prepared supramolecular nanocapsules consisted of QDs conjugated with polyhistidine-tagged proteins and attached electrostatically with cationic Au NPs, these being Au NPs pinned to the capsule surface of an oil core [37]. These nanocapsules demonstrated multifunctional properties in cellular studies, such as fluorescent tracking of intracellular bioconjugates and stimuli-responsive protein delivery. Another interesting example was reported by Algar et al., who prepared Ab-conjugated supra-QD assemblies for selective immunolabeling of breast cancer cells [39]. These supra-QD assemblies that comprised many QDs around a central silica nanoparticle (SiO_2_@QDs) and tetrameric antibody complexes (TACs) conjugated to the QDs surface through dextran molecules. Despite the smart design and good performance of these aforementioned NP assemblies, the synthetic procedures behind are not straightforward; they involve several types of NPs and multiple conjugation reactions to connect the different parts of the entire nanoassembled entity, which might somewhat dampen further developments in their biological applications in more realistic scenarios. Another clinical area where QD nanoassemblies are making a significant impact is in the development of bioassays such as fluorescent lateral flow assays (fLFA) [40,41,42]. As a recent example, multi-quantum dots embedded in silica-encapsulated nanoparticles were functionalized with anti-CD63 Abs and were applied to fLFA to sensitively detect human exosomes, a biomarker for certain diseases [41]. Although antibody-conjugated QD nanoassemblies reported to date, most of them by integrating QDs with silica or polymer matrices, can be very useful for biological assays, their large size (>200 nm) may be a drawback for intracellular or in vivo applications [43]. These are just some examples that illustrate the present growing importance of QD nanoassemblies in the area of bioapplications.

With the aim of contributing to this field, we previously developed a simple strategy for preparing highly robust fluorescent 50 nm-nanoassemblies of QDs driven only by fluorine–fluorine hydrophobic interactions [36]. Herein, we wanted to go a step further by demonstrating that Abs could be also conjugated to such bigger NP assemblies through a simple but controlled functionalization approach, and that in vitro targeting could be possible not only with QDs but also with these QD assemblies of bigger size. To demonstrate this, we prepared fluorescent antibody-conjugated fluorinated QD nanoassemblies (immune-QDassemblies) and performed cell uptake studies.

## 2. Materials and Methods

### 2.1. Preparation of NP Assemblies Conjugated to Antibodies 

The here prepared and studied NP assembly consist of fluorescent core/shell CdSe/ZnS quantum dots (QDs) modified with fluorinated and carboxylated ligands for their further assembly and functionalization. Fluorinated ligands allowed the self-assembly of the QDs into nanoassemblies (QD NAs) of about 50 nm in diameter, whereas caraboxylated ligands provide the reactive groups for the further attachment of antibodies (i.e., anti-CD44 mAb) by means of 1-ethyl-3-(3-dimethylaminopropyl)carbodiimide (EDC) chemistry. All the synthetic procedures are described in detail in the Supplementary Materials.

### 2.2. Characterization Techniques 

Physico-chemical characterization of the NP assemblies before and after the attachment of antibodies was done using transmission electron microscopy (TEM), UV/Vis absorption spectroscopy, fluorescence spectroscopy, nuclear magnetic resonance (NMR) spectroscopy, dynamic light scattering (DLS), laser Doppler anemometry (LDA), and inductively coupled plasma mass spectrometry (ICP-MS). In order to estimate the number of antibodies per nanoassembly and the surface coverage, the amount of antibodies on the QD NAs was measured using the bicinchoninic acid (BCA) protein assay. All protocols are described in detail in the Supplementary Materials.

### 2.3. Cell Studies 

We confirm that the biological material involved in the study (human breast cancer cell line—MDA-MB-231, and mouse embryonic fibroblast cell line—NIH 3T3) are readily available from standard commercial source (ATCC). A standard experiment consists of incubation of cells with the QD NAs or mAb-QD NAs at different concentrations (5 nm–50 nm) for a specific period of time (1 h–6 h). In all the cell studies, non-internalized particles were washed out from the cell culture before analysis. The cytotoxicity of the NAs was measured with the colorimetric (3-(4,5-dimethylthiazol-2-yl)-2,5-diphenyltetrazolium bromide) assay (MTT assay). Cellular uptake studies of the NAs were performed by fluorescence imaging using a confocal laser scanning microscope (CLSM) and by flow cytometry (FC) analyses. The CD44 expression levels in the MDA-MB-231 and NIH 3T3 cell lines were evaluated by immunoblotting analysis with reference to β-actin. Detailed protocols can be found in the Supplementary Materials.

## 3. Results and Discussion

### 3.1. Design, Preparation, and Physicochemical Characterization of Anti-CD44 mAb-Conjugated QD Nanoassemblies

As a model antibody, we chose an anti-CD44 monoclonal antibody (mAb), which has widely demonstrated both in vitro and in vivo its potential as an efficient targeting agent toward CD44-overexpressing cells alone [44], as well as after being conjugated to NPs [45,46]. CD44 is a trans-membrane glycoprotein that has been identified to be overexpressed in several cancer types including breast, pancreas, prostate, colon, and gastric cancer [47,48]. Concerning the NPs, we selected QDs owing to their intrinsic fluorescence [49]. In vitro cell studies by using confocal laser scanning microscopy (CLSM) and flow cytometry (FC) were accomplished to probe cellular internalization of the QD nanoassemblies in cells with different CD44 expression levels.

**Figure 1:**
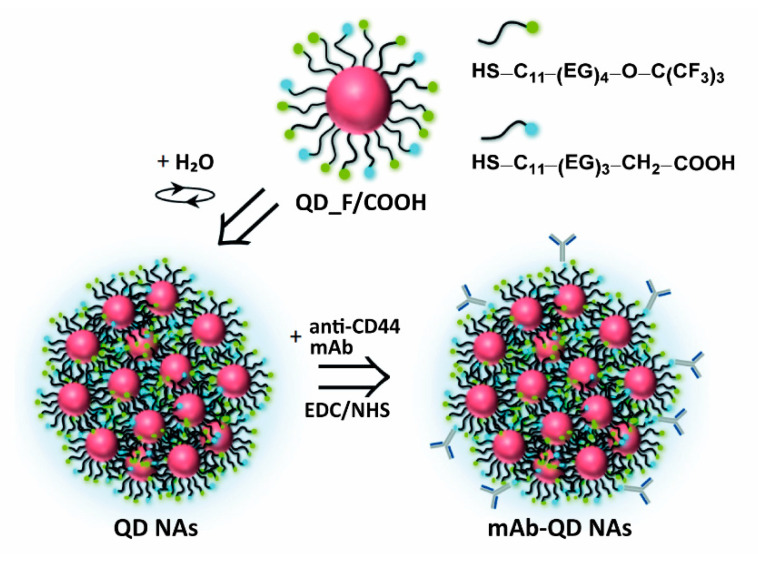
Scheme of the preparation of the mAb-QD NAs based on the self-assembly of QD_F/COOH nanoparticles for obtaining first QD nanoassemblies (QD NAs), and their posterior functionalization with anti-CD44 mAb via EDC chemistry resulting in mAb-QD NAs.

The mAb-conjugated fluorinated QD nanoassemblies (in the following referred to as mAb-QD NAs) were synthesized in three steps (Figure 1). First, core-shell CdSe/ZnS QDs with an inorganic core/shell diameter of d_QD_ ~5 nm, capped with trioctylphosphine oxide (TOPO) (Figure S1A), were synthesized through the hot injection method, and their surface was further modified to incorporate fluorine atoms and carboxylic groups by using a ligand exchange approach [36]. This consisted of replacing the hydrophobic TOPO ligands introduced during the QD synthesis with a mixture of fluorinated (HS–C_11_–(EG)_4_O–C(CF_3_)_3_) and carboxylated ligands (HS–C_11_–(EG)_3_–CH_2_–COOH), in a 9:1 molar ratio, which was optimized to have sufficient carboxylic groups for the conjugation of the antibody but maintaining the maximum fluorinated groups for the self-assembly of the QDs (see Supporting Information for details). The resulting QD_F/COOH nanoparticles presented an inorganic core/shell diameter d_QD_ of (5.0 ± 0.8) nm, first exciton absorption peak at 582 nm, and maximum fluorescence emission peak at 603 nm (Figure S1E,F). Ligand modification of the QDs was investigated by ^1^H NMR and ^19^F NMR (Figure S2). Unfortunately, the presence of some unbound fluorinated molecules, together with the low amount of carboxylated ligands incorporated onto the QD surface (below the detection limit of the NMR set-up, thus making thus the protons from COOH groups not visible), did not allow for the unequivocal confirmation of the QD functionalization. Neverthless, despite this limitation, the further formation of the nanoassemblies built from QD_F/COOH undoubtedly indicated the presence of the added ligands on the QD surface, since the non-modified QDs did not lead to their self-assembly. Moreover, for the targeting experiments with the final Ab-modified nanoassemblies, the potential presence of some free ligands with the QD_F/COOH is not crucial, as, upon the formation of nanoassemblies from QD_F/COOH and the following antibody conjugation, there are several additional purification steps involved.

**Figure 2:**
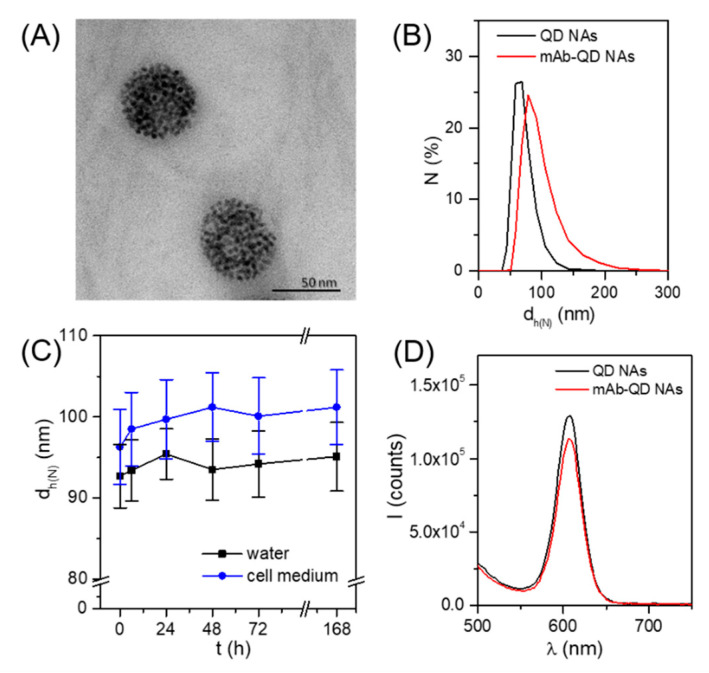
(**A**) TEM image of the QD NAs with a mean diameter of d_QD NA_ = (58 ± 7) nm; (**B**) DLS number distributions of the hydrodynamic diameter (d_h_) of mAb-QD NAs (d_h_ = 93 ± 4 nm; polydispersity index PDI = 0.25) and non-modified QD NAs (d_h_ = 68 ± 3 nm; PDI = 0.21); (**C**) colloidal stability in terms of hydrodynamic diameter over time of mAb-QD NAs in both water and cell medium; (**D**) fluorescence emission spectra of the mAb-QD NAs and non-modified QD NAs under excitation at 405 nm.

The second step consisted of the self-assembly of the QDs driven by fluorine–fluorine hydrophobic interactions, as previously reported [36]. The key factor for the effective self-assembly of QDs in aqueous solution as well as the robustness of the obtained NAs relies on the high amount of fluorine atoms per ligand, thus allowing multiple hydrophobic fluorine–fluorine interactions between the QDs. Briefly, QD_F/COOH dispersed in DMSO were mixed with water to a final ratio (*v*/*v*) of DMSO:H_2_O 5:95, stirring vigorously for 1 min, and were left undisturbed for 10 min for stabilization. Such a simple procedure led to homogeneous QD NAs with a mean diameter d_QD NA_ of (58 ± 7) nm as determined by transmission electron microscopy (TEM, Figure 2A and Figure S1C), and having a hydrodynamic diameter d_h_ of (68 ± 3) nm according to dynamic light scattering (DLS) analysis (Figure 2B and Table S1). These QD NAs were negatively charged, having a zeta potential of (−42 ± 2) mV. Note that this value was slightly superior to the NAs obtained from QD_F without containing carboxylated ligands, i.e., (−35 ± 1) mV [36], which was expected due to the presence of carboxylic groups on their surface that are likely to be deprotonated at the pH of Milli-Q water (*ca.* 6.5). Moreover, this more negative surface charge gave us the confirmation that the carboxyl groups were exposed to the outer surface, and, therefore, would be accessible for their posterior functionalization. Finally, anti-CD44 mAbs, which were selected in this work as targeting molecules, were attached to the QD NAs via EDC chemistry by reaction of the primary amine groups of the mAb with the carboxylic groups on the surface of the QD NAs (see Supporting Information for details). The obtained mAb-QD NAs presented an increase of the size (hydrodynamic diameter d_h_ = 93 ± 4 nm, Figure 2B and Table S1) and a decrease of the negative charge (ζ-potential of −25 mV) in comparison to the non-modified QD NAs, which suggests attachment of the targeting mAb molecules. Both a moderate size increase and sharp distribution of the hydrodynamic diameter did not indicate aggregation of the NAs during the conjugation of the mAb, which is important concerning the analysis of the cellular uptake studies [50]. Moreover, this size increase (~25 nm) would be consistent with a single layer of mAb [51]. Importantly, these mAb-QD NAs were colloidally stable in aqueous solution, as well as in supplemented cell medium (Figure 2C) for at least one week, as demonstrated by DLS. 

It is important to note that, following this synthetic procedure, the mAbs are bound covalently to the surface of the NAs and not encapsulated inside as proposed in our previous work with other proteins [36]. Here, the attachment of the mAbs involves a post-functionalization step on the already formed NAs. On the contrary, if the self-assembly process of the QDs takes place in the presence of proteins (e.g., enzymes [36]), previously adsorbed on the surface of individual QDs, the majority of these proteins are finally encapsulated within the NAs by physical entrapment (although it cannot be ruled out that some proteins remain adsorbed on the outside of the NAs). The amount of mAbs immobilized on the NA surface was quantified by the BCA protein assay (see Supporting Information for details), giving that roughly on average ~143 mAb molecules were attached per QD NA. Based on the surface footprint of Y-shaped antibody molecules [52], this would correspond to between 19% and 43% (depending on the mAb orientation) of the QD NA surface covered by mAb. We note that, in the present study, no experimental data were obtained about the orientation of the attached Abs, neither on potential deactivation of the active sites upon conjugation.

Ligand exchange and conjugation procedures may alter the optical properties of QDs, e.g., due to agglomeration. As can be seen in Figure 2D, the characteristic fluorescence of the QD NAs was fairly well preserved after the mAb conjugation, both in terms of intensity and narrow width of the emission band. This is in agreement with the DLS measurements in which no significant agglomeration was detected.

### 3.2. Cellular Uptake of Anti-CD44 mAb-Conjugated QD Nanoassemblies 

Uptake studies of the prepared mAb-QD NAs were performed with human breast carcinoma cells (MDA-MB-231) that present CD44 overexpression (CD44^+^). Western blot analysis demonstrated the high expression level of CD44 in the MDA-MB-231 cell line as compared to a control cell line; mouse embryonic fibroblast cells (NIH 3T3) with a lack CD44 expression (CD44^−^) were selected as control cells (Figure S3). For the uptake studies of the mAb-QD NAs, first their cytotoxicity was studied by the standard MTT assay in a range of concentrations (from 5 nm to 50 nm, expressed as QD concentration) and at different incubation times (3 h, 6 h, and 12 h). Results revealed that, while there is the known Cd-induced toxicity of the QDs, incubation for 6 h at 10 nm of mAb-QD NAs still maintained ca. 85% of viability of the MDA-MB-231 cells (Figure S4). In this condition, there was already significant uptake of the NAs, resulting in strong fluorescence signals in microscopy and cytometry studies. Longer incubation times and/or higher concentrations resulted in much higher internalization rates, and consequently in higher cytotoxicity.

**Figure 3:**
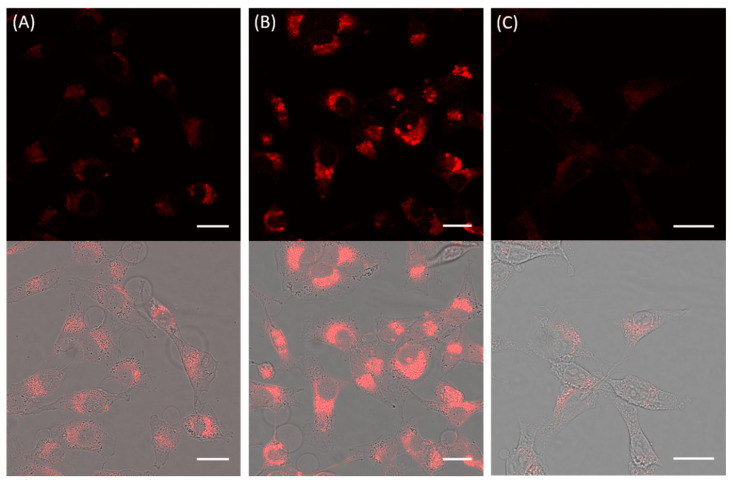
Representative confocal microscopy images of cells incubated with modified and non-modified QD NAs (10 nm, 6 h, 37 °C): (**A**) MDA-MB-231 cells treated with QD NAs; (**B**) MDA-MB-231 cells treated with mAb-QD NAs; and (**C**) MDA-MB-231 cells pre-treated with free anti-CD44 mAb molecules (2 µg/mL, incubation for 1 h at 37 °C), and treated further with mAb-QD NAs. Fluorescence was collected using a LP 560 emission filter under excitation at 405 nm and using a 63× objective. In the upper line, the fluorescence channel with the fluorescence of the QD NAs displayed in red is shown, and is merged with the transmission channel as shown in the lower line. The scale bars correspond to 25 μm.

Under conditions of tolerable toxicity (10 nm, 6 h incubation at 37 °C), the internalization of mAb-QD NAs was visualized by confocal laser scanning microscopy (CLSM). Figure 3A shows that MDA-MB-231 (CD44^+^) cells showed under the given exposure condition only moderate uptake of QD NAs without attached antibody. The presence of anti-CD44 mAb strongly increased the uptake of the mAb-QD NAs (Figure 3B). It is unlikely that this change was caused only by the size difference between mAb-QD NAs and QD NAs (d_h_ of 93 nm versus 68 nm). The uptake mechanism apparently occurred via endocytic pathway, as revealed by the resulting punctate fluorescence, with QD NAs being localized mostly in endosomes. Furthermore, in the case of mAb-QD NAs, it seems that bigger aggregates were formed within the endosomes, which are seen as large dots with more intense fluorescence. 

To further confirm that the increased uptake of mAb-QD NAs by CD44^+^ cells was mediated through the interaction of anti-CD44 mAb with CD44 receptors on cell surfaces, a competitive binding experiment was performed by pre-treating the MDA-MB-231 cells with a saturable amount of free anti-CD44 mAbs (2 µg/mL, incubation for 1 h at 37 °C) before the incubation with mAb-QD NAs. As depicted in Figure 3C, the cellular uptake of mAb-QD NAs was effectively reduced in comparison to cells with non-blocked receptors (Figure 3B), indicating that the free mAbs compete with the mAb-QD NAs for receptor binding sites, and therefore confirming the CD44 receptor-mediated endocytosis. Besides this finding, the uptake of mAb-QD NAs by NIH 3T3 (CD44^−^) cells led to a meaningful amount of fluorescence coming from internalized mAb-QD NAs (Figure S5). However, the results for MDA-MB-231 (CD44^+^) and NIH 3T3 (CD44^−^) cannot be directly compared, as in general cells have different uptake kinetics also in the case of nonspecific uptake. Additional flow cytometry (FC) experiments showed that uptake of mAb-QD NAs followed the typical time-dependent increase (Figure S6).

## 4. Conclusions

Altogether, the main findings of this study can be summarized as follows: (i) mAb molecules could be successfully immobilized on the surface of assembled NPs, and, importantly, this did not affect the photoluminescence properties of the QDs; and (ii) a sufficient fraction of immobilized mAb molecules retained their targeting ability. Even if we cannot rule out some loss of targeting ability, due to the presence of many mAbs per QD, NA targeting is possible. (iii) The prepared nanoassemblies were uptaken by cells in a time-dependent manner known for endocytosis. The presence of the mAb molecules could add specific receptor-ligand based endocytosis on top of nonspecific uptake. While this is a qualitative and not quantitative study, it could be demonstrated that antibody-mediated uptake is also possible for nanoparticle assemblies.

## Data Availability

The datasets generated during and/or analyzed during this study are not publicly available but are available from the corresponding author upon reasonable request.

## Supplementary Materials

The following are available online at https://www.mdpi.com/article/10.3390/nano11081906/s1; Experimental details of synthetic methods, functionalization procedures, characterization techniques, cell procedures, and imaging and cytometry protocols. Figure S1: Physico-chemical characterization of QD NAs; Figure S2: ^1^H NMR and ^19^F NMR spectra of modified QDs; Table S1: DLS measurements of QD NAs and mAb-QD NAs; Figure S3: Western blot analysis of CD44 expression in MDA-MB-231 and NIH 3T3 cells; Figure S4: Cell viability upon incubation with mAb-QD NAs; Figure S5: Representative confocal microscopy images of cells incubated with mAb-QD NAs; Figure S6: Flow cytometry analyses.
