# Supplementary material for: In Vitro Cellular Uptake Studies of Self-Assembled Fluorinated Nanoparticles Labelled with Antibodies
Source: Nanomaterials (Basel). 2021 Jul 24;11(8):1906. doi: 10.3390/nano11081906 (PMC8401737; doi:10.3390/nano11081906)
Supplement: Supplementary file 1 [file nanomaterials-11-01906-s001.zip › nanomaterials-1280489-supplementary.pdf]

nanomaterials

MDPI

Supplementary Materials

In Vitro Cellular Uptake Studies of Self-Assembled Fluorinated Nanoparticles Labelled with Antibodies

Mona Atabakhshi-Kashi [1,2], Mónica Carril [2,3,4,5], Hossein Mahdavi [6], Wolfgang J. Parak [2,7], Carolina Carrillo-Carrion [2,8,*], and Khosro Khajeh [1,*]

1 Department of Nanobiotechnology, Tarbiat Modares University, Tehran 14115-175, Iran; monaatabakhshi@gmail.com
2 Bioengineered Particles Group, CIC biomaGUNE, 20014 San Sebastian, Spain; monica.carrilg@ehu.eus (M.C.); wolfgang.parak@uni-hamburg.de (W.J.P.)
3 Ikerbasque, Basque Foundation for Science, 48013 Bilbao, Spain
4 Instituto Biofisika UPV/EHU, CSIC, 48940 Leioa, Bizkaia, Spain
5 Departamento de Bioquímica y Biología Molecular, UPV/EHU, 48940Leioa, Bizkaia, Spain
6 School of Chemistry, College of Science, University of Tehran, Tehran 14174-66191, Iran; hosinmahdavi@ut.ac.ir
7 Fachbereich Physik and CHyN, Universität Hamburg, 22607 Hamburg, Germany
8 Institute for Chemical Research (IIQ), CSIC-University of Seville, 41092 Sevilla, Spain
* Correspondence: carolina.carrillo@csic.es (C.C.-C.); khajeh@modares.ac.ir (K.K.)

1. Synthetic Procedures

1.1. Chemicals and Biochemical Reagents

Cadmium oxide (CdO, #202894), hexylphosphonic acid (HPA, #750034), trioctylphosphine oxide (TOPO, #223301), trioctylphosphine (TOP, #718165), selenium powder (Se, #229865), diethylzinc solution ($ZnEt_2$ 1 M in hexane, #296112), hexamethyldisilathiane ($(TMS)_2S$, #283134), N-(3- dimethylaminopropyl)-N′- ethylcarbodiimide hydrochloride (EDC, #03449), N-Hydroxysuccinimide (NHS, #13072), and 3-(4,5-dimethylthiazol-2-yl)- 2,5-diphenyltetrazolium bromide (MTT, M2128) were obtained from Sigma-Aldrich (Darmstadt, Germany). 2-Morpholinoethanesulfonic acid monohydrate (MES, #106126**)** was obtained from Merck Company (Darmstadt, Germany). A human breast cancer cell line (MDA-MB-231) and mouse embryo fibroblast cells (NIH 3T3) were obtained from American type culture collection (ATCC). CD44 monoclonal antibody (#MA5-13890) was purchased from Thermo Fisher Scientific. Goat anti-mouse IgG HRP conjugate (#ab97023), β-Actine antibody (#ab8227), and the BCA protein assay kit (#ab103526) were purchased from Abcam (Cambridge, UK). 10 and 300 kDa centrifugal filter device (#OD010C33 and #OD300C33) were purchased from Pall Corporation (Hampshire, UK). Penicillin- streptomycin (#15140148), fetal bovine serum (FBS, #10082147), trypsin-EDTA (0.25%) (#25200056), and Dulbecco's Modified Eagle high glucose medium (DMEM, #11965092) were purchased from Gibco (Thermo Fisher Scientific, Carlsbad, CA, USA)

1.2. Quantum Dot Synthesis

Fluorescent core-shell CdSe/ZnS quantum dots (QDs) capped with trioctylphosphine oxide (TOPO) with a core diameter of 5 nm were synthesized through the hot injection method in two steps. First, the cores of CdSe were synthesized using cadmium oxide as a precursor. Secondly, the ZnS shells were deposited on CdSe via a previously reported synthetic protocol [1].

1.3. Ligands

The fluorinated ligand HS–C_{11}–$(EG)_4$–O–C$(CF_3)_3$ and non-fluorinated carboxylated ligand HS–C_{11}– $(EG)_3$–CH_2–COOH were synthesized as already reported in previous articles [2,3].

1.4. Preparation of Fluorinated/Carboxylated Quantum Dots

Fluorinated/carboxylated quantum dots (QD_F/COOH) were prepared through a slight modification of a previously reported ligand exchange protocol for the synthesis of fluorinated QDs (QD_F) [4]. Briefly, the fluorinated ligand (2.07 mL, 90 µmol) and the carboxylated ligand (0.23 mL, 10 µmol) were mixed (in a 1:9 molar ratio), then added to 2.5 mL of dichloromethane (DCM) and 5 mL of methanol (MeOH) in a glass vial. TOPO-capped QD solution (0.6 mL, 5 nmol) was then added, and the mixture was stirred overnight in darkness at 40 °C. The next day, the QD_F/COOH were collected by centrifugation ($1.5x10^5$ rcf, 30 min). The pellet was redispersed in 0.5 mL of DCM, 3 mL of MeOH were added, and the particles were collected again by centrifugation ($1.5x10^5$ rcf, 30 min). This process was repeated three times, and finally the purified QD_F/COOH were dispersed in DCM and stored in darkness until use.

The concentration of QDs in terms of nanoparticle concentration, regardless of the nature of the organic ligands on their surface (e.g., TOPO, fluorinated or carboxylated compounds), was determined by inductively coupled plasma mass spectrometry (ICP-MS) as follows [1]. The QD sample was subjected to acidic digestion with aqua regia (3:1 mixture of hydrochloric acid and nitric) overnight, and then appropriately diluted in Milli-Q water for further analysis. External calibration with standard solutions (obtained from Agilent) was applied to quantify the amount of elemental cadmium (Cd) and zinc (Zn). From ICP-MS analysis we obtained these concentrations in mg/mL; C_{Cd} is the concentration of Cd (corresponding to CdSe) and C_{Zn} is the concentration of Zn (corresponding to ZnS). Taking into account that the molar stoichiometry Cd:Se is assumed to be 1:1 in the NP core, and in the same way the molar stoichiometry Zn:S is assumed to be 1:1 in the NP shell, the mass of elemental selenium (Se) and sulfur (S) in solution can be calculated as:

$$m_{Se} = m_{Cd} \cdot \frac{M_{Se}}{M_{Cd}} \tag{1}$$

$$m_{S} = m_{Zn} \cdot \frac{M_{S}}{M_{Zn}} \tag{2}$$

hereby M_{Cd}, M_{Zn}, M_{Se} and M_S are the atomic weights of each element.

The mass of all QDs in solution (neglecting the mass of the organic ligands) is therefore:

$$m_{QD} = m_{Cd} + m_{Se} + m_{Zn} + m_{S}, \tag{3}$$

Assuming that the QDs are spherical, and using the mean diameter obtained by TEM (d_{QD} = 5.0 nm), the volume of an individual QD is determined with the following equation:

$$V_{QD} = \left(\pi/6\right) \cdot d_{QD}^3 \tag{4}$$

The total volume of QDs ($V_{t,QD}$) in solution can be calculated as:

$$V_{t,QD} = m_{QD}/\rho_{QD} \tag{5}$$

The bulk densities of CdSe and ZnS are known (ϱ_{CdSe} = 5.82 g/cm^3 and ϱ_{ZnS} = 4.09 g/cm^3), the density of the core-shell CdSe/ZnS structure (ρ_{QD}) has to be estimated by taking the densities of CdSe and ZnS weighted by their mass fractions:

$$\rho_{QD} = \rho_{CdSe} \cdot \frac{C_{Cd}}{C_{QD}} + \rho_{ZnS} \cdot \frac{C_{Zn}}{C_{QD}} \quad (6)$$

hereby C_{Cd} and C_{Zn} are the concentrations obtained by ICP-MS in mg/mL of Cd (corresponding to CdSe) and Zn (corresponding to ZnS), respectively, while C_{QD} corresponds to the mg/mL of the total CdSe/ZnS QD and it is calculated as $C_{QD} = C_{Cd} + C_{Zn}$.

The total number N_{QD} of QDs in solution can be calculated as:

$$N_{QD} = {}^{V_{t,QD}}\!/_{V_{QD}} \quad (7)$$

And finally, the molar concentration c_{QD} of QDs can be calculated as:

$$c_{QD} = \frac{{}^{N_{QD}}\!/_{N_A}}{V} \quad (8)$$

where N_A is the Avogadro constant and V is the total volume of the QD sample solution.

1.5. Preparation of Quantum Dot Nanoassemblies (QD NAs)

To be in a solvent miscible with water the QD_F/COOH were transferred from DCM to dimethylsulfoxide (DMSO). Then, 50 μL of the QD_F/COOH solution (2 μM; particle concentration determined by ICP-MS as described above) in DMSO was added to 950 μL of Milli-Q water or a buffer solution, and the mixture was stirred vigorously for 1 min with a vortex mixer and left to stand 10 min for stabilization. The concentration of the QDs in the final sample was 100 nM, and they were dispersed in a medium DMSO:H_2O (5:95 v/v).

1.6. Functionalization of QD NAs with Anti-CD44 Monoclonal Antibody (anti-CD44 mAb)

QD NAs were conjugated to CD44 monoclonal antibody via amide bond formation between primary amines on the mAb and carboxylic groups on the QD surface. For that, 1 mL of as-prepared QD NAs at 100 nM in MES buffer (100 mM, pH 6) were mixed with 0.5 mL of 2 mM EDC and 8 mM NHS, and were incubated for 20 min at room temperature with gentle mixing. The remaining unreacted reagents were removed by centrifugation using a 10 kDa centrifugal filter device (1×10^3 RCF, 5 min). The activated QD NAs were then resuspended in 1mL of HEPES buffer (100 mM, pH 8.5):DMSO (95:5) which contained 30 μg mL^{-1} antibody solution to obtain 2-fold molar excess of the mAb to QDs. The reaction mixture was incubated for 3 h at room temperature under the gentle stirring. Excess of reagents and unconjugated mAb were removed by washing three times in 300 kDa centrifugal filter devices. The purified mAb-QD NAs were finally dispersed in DMSO:H_2O (5:95 v/v) at a concentration of 0.1 μM for characterization or in DMSO:phosphate buffered saline (PBS) (5:95 v/v) at a concentration of 0.5 μM for performing cell experiments.

2. Characterization Techniques

2.1. Transmission Electron Microscopy (TEM)

The morphology and size of the QD_F/COOH and QD NAs was investigated by TEM by deposition of the sample on top of carbon-coated copper grids followed by drying at environmental conditions. Images were acquired with a JEOL JEM 2100F microscope (JEOL GmbH, Freising, Germany) operated at 200 kV accelerating voltage. The free software ImageJ and the software Origin were used for the size measurements from the TEM images and distribution analysis, respectively (Figure S1).

2.2. UV/Vis Absorption Spectroscopy

Absorption spectra were acquired with a Varian 5000 UV-Vis-NIR spectrophotometer using a 10 mm path length glass cuvette (Figure S1).

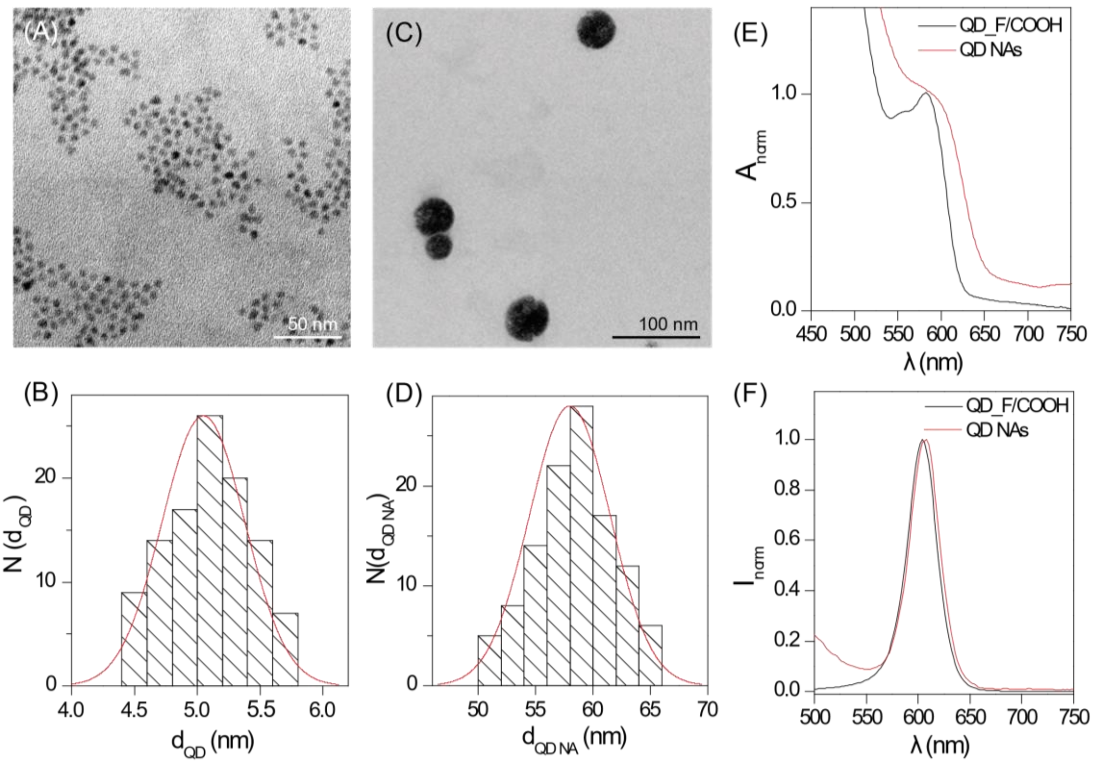

Figure S1. (**A**) TEM image of QD_F/COOH, and (**B**) the corresponding histogram of the size distribution of the inorganic core diameter N(d_{QD}) with mean diameter d_{QD} = 5.0 ± 0.8 nm. (**C**) TEM image of QD NAs, and (**D**) the corresponding histogram of the size distribution of the inorganic core diameter N($d_{QD\ NA}$) with mean diameter $d_{QD\ NA}$ = 58 ± 7 nm. N(d) refers to the total counts. (**E**) Normalized absorption spectra of QD_F/COOH and QD NAs. (**F**) Normalized fluorescence spectra of QD_F/COOH and QD NAs under excitation at 405 nm.

2.3. Fluorescence Spectroscopy

Fluorescence emission spectra of the samples were collected using a Fluorolog-3 (model FL3-22, Horiba) fluorescence spectrometer. Measurements were carried out using a 10 mm path length quartz cuvette. Fluorescence spectra were recorded under excitation at 405, and the integration time was set to 0.1 s per 2 nm increment in wavelength (Figure S1).

2.4. Nuclear Magnetic Resonance (NMR) Spectroscopy

^{1}H and ^{19}F NMR spectra were recorded in CD_2Cl_2 solution in a Bruker 500 MHz spectrometer (500 MHz for ^{1}H and 470 MHz for ^{19}F), see Figure S2. Spectra were analyzed with the software MestReNova. Unfortunately, there can't be definite statements drawn from the spectra, because of complications with the purification of QD_F/COOH. The signals at 2.5 and 2.7 ppm show that those QDs were not clean. The signals there belong to unbound fluorinated ligand, not to ligands on the QD surface. In addition, the COOH ligand is not visible. This could be for several reasons. On the one hand, there might have been so much free ligand that it did not allow to see what was attached to the surface of the QDs. On the other hand, the amount of carboxylated ligand might have been so small that was below the sensitivity of the NMR set-up. While formation of the nanoassemblies built from QD_F/COOH suggests the presence of the added ligands on the QD surface, from the here shown NMR spectra the functionalization of the QDs cannot be proven. For the targeting experiments with antibody modified nanoassemblies the presence of free ligands upon formation of QD_F/COOH is not crucial, as upon the formation of nanoassemblies from QD_F/COOH and the following antibody conjugation there are several additional purification steps involved.

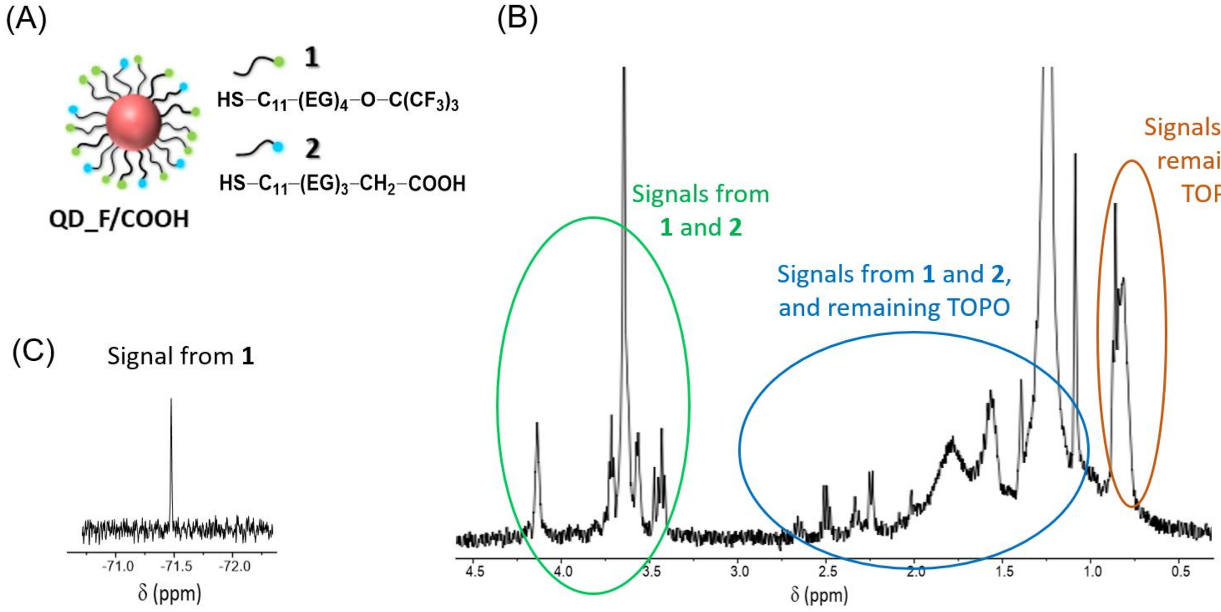

Figure S2. (**A**) Scheme of QD containing fluorinated (1) and carboxylated (2) ligands. (**B**) ^{1}H NMR spectrum of QD_F/COOH nanoparticles showing the chemical shift δ. (**C**) ^{19}F NMR spectrum of QD_F/COOH. All spectra were recorded in CD_2Cl_2.

2.5. Dynamic Light Scattering (DLS) and Zeta-potential

A Malvern Zetasizer (Nano ZS ZEN3600), equipped with a 10 mW He–Ne laser operating at a wavelength of 633 nm and fixed scattering angle of 173° was used to measure the hydrodynamic diameters (with dynamic light scattering, DLS, cf. Table S1), and the zeta-potential (with laser Doppler anemometry, LDA). Measurements were done placing the samples in a glass cuvette and the specific parameters of temperature and refractive index and viscosity of the medium (DMSO:H_2O 5:95 v/v) were fixed as follows: temperature = 25 °C, refractive index: 0.96; viscosity: 1,3390 cP.

Table S1. Hydrodynamic diameters d_h (mean value ± SD) from DLS intensity (I), volume (V), and number (N) distributions from DLS measurements of the QD NAs and mAb-QD NAs. The polydispersity index (PDI) for each sample is also given.

Sample	$d_{h(I)}$ (nm)	$d_{h(V)}$ (nm)	$d_{h(N)}$ (nm)	PDI
QD NAs	103 ± 7	92 ± 5	68 ± 3	0.21
mAb-QD NAs	132 ± 8	121 ± 7	93 ± 4	0.25

3. Estimation of the Number of Antibodies per Nanoassembly and Surface Coverage

3.1. Determination of Immobilized mAb Molecules

The amount of immobilized antibodies on the QD NAs was measured using the bicinchoninic acid (BCA) protein assay kit. In this procedure, 50 µL of mAb-QD NA sample (at a concentration of 100 nM in terms of QDs) was combined with 100 µL of BCA reagents in triplicate, and left to react in a water bath at 37 °C for 45 min. After that, the absorbance of the mixture was measured at 562 nm, and the net absorbance was calculated by subtracting the obtained absorbance signal of non-modified QD NAs (as blank). A calibration curve was prepared by plotting the absorbance at 562 nm of standard solutions of bovine

serum albumin versus its concentration, and fitting to a linear equation. By using the calibration equation, the amount of mAb protein in 1 mL of mAb-QD NAs sample (containing 1×10^{-10} moles of QDs) was calculated to be 2.38 μg. Applying the molecular weight of anti-CD44 mAb ($M_W = 1.5\times10^5$ g/mol), the total number of mAb in the sample was 9.55×10^{12}.

3.2. Estimation of the Number of mAb per QD NA (mAb/QD NA ratio)

To determine the mean number of immobilized mAb onto the surface of each NA, we first calculated the number of QDs per NA and then determined the number of NAs in 1 mL of mAb-QD NA sample. Based on previously reported equations [4], the number of QDs per NA ($N_{QD/NA}$) can be estimated by the following equation:

$$N_{QD/NA} = (d_{QD\ NA}/d'_{QD})^3 \quad (9)$$

where $d_{QD\ NA}$ determines the diameter of one NA ($d_{QD\ NA}$ ~58 nm) and d'_{QD} determines the effective diameter of one QD ($d'_{QD} = d_{QD}$ + 1 nm ~6 nm, whereby the 1 nm accounted for the organic ligands on the QD surface) as derived from TEM analysis. Applying this equation, one NA is composed of approximately 903 QDs, whereby this has to be seen as upper limit, in which the QDs would be packed at maximum density. By knowing the total number of QDs per 1 mL sample (1×10^{-10} moles, which using the Avogadro's number corresponds to 6.02×10^{13} QDs) the number of NAs per 1 mL of the sample is estimated to be around 6.67×10^{10}.

Knowing the number of mAb per sample (calculated above), the mAb/QD NA ratio resulted to be 143.

3.2. Antibody Surface Coverage

Assuming that the NAs are spherical, their surface area ($A_{QD\ NA}$) can be determined using the following equation:

$$A_{QD\ NA} = 4\pi\ (d_{QD\ NA}/2)^2 \quad (10)$$

Using the diameter of one NA ($d_{QD\ NA}$ ~58 nm), the surface area of one NA is thus 10568 nm^2. The surface footprint of the Y-shaped IgG molecule is between 14 and 32 nm^2 [5], depending on the antibody orientation. Based on the mAb size and knowing that the number of mAb molecules per QD NA is 185, this would result in a surface coverage of ~2002 nm^2 (~19% of the NA surface covered by mAb molecules) for end-on orientation (*i.e.* vertically oriented), and ~4576 nm^2 (~43% of the NA surface covered by mAb molecules) for side-on orientation (*i.e.* horizontally oriented).

4. Cell Studies

4.1. Cell Culture

All cell lines were cultured at 37 °C and 5% CO_2 in 75 cm^2 flasks in complete medium, i.e. Dulbecco's modified Eagle's medium (DMEM) supplemented with 10 % heat-inactivated fetal bovine serum (FBS), 2 mM L-glutamine, 100 μg/mL streptomycin, and 100 U/mL penicillin. Cells were routinely passaged by treatment with trypsin-EDTA (0.25%). Cell counting was performed by using the exclusion dye Trypan Blue in a haemocytometer chamber under transmitted light in an inverted microscope (DMIL, Leica Microsystems GmbH, Wetzlar, Germany).

4.2. Western Blot Analysis

The CD44 expression levels in the MDA-MB-231 and NIH 3T3 cell lines were evaluated by immunoblotting analysis with reference to β-actin. Immunoblotting analysis was performed using specific antibodies for CD44 and β-actin. For whole cell protein extraction, approximately 10^7 cells were washed twice in precooled PBS and lysed in 400 μL of

radioimmunoprecipitation assay (RIPA) cell lysis buffer, which was prepared based on the Abcam protocol containing protease inhibitors and 1% Triton X-100. After incubation for 30 min at 4 °C, the cell lysate was centrifuged at 15×10^3 RCF for 10 min, and the supernatant was analyzed for protein content using the Bradford protein assay protocol. In the following, an equal amount (50 µg) of total protein were subjected to 12% sodium dodecyl sulfate–polyacrylamide gel electrophoresis (SDS-PAGE). Subsequently, proteins were separated and transferred on to the polyvinylidene difluoride (PVDF) membranes (Roche Life Science). The blot was incubated in blocking solution which contain 5% [w/v] of bovine serum albumin (BSA) in TBS/T buffer (200 mM Tris base (pH 7.6), 150 mM NaCl, and 0.1% Tween 20) for 2 h at room temperature. The blocked blots were incubated overnight with primary antibodies using 2 $\mu g\cdot mL^{-1}$ of CD44 antibody dilutions in TBS/T buffer. After several washing steps, the PVDF membrane was incubated with anti-mouse horseradish peroxidase (HRP) conjugated secondary antibody. Detection of protein was performed by an enhanced chemiluminescence system. The membrane was re-probed with β-actin antibody for protein normalization. Immunoblotting analysis as depicted in Figure S3 clearly indicated that, compared to the control NIH 3T3 cell line, the MDA-MB-231 cell line expressed high levels of CD44. This difference in CD44 expression level between MDA-MB-231 and NIH 3T3 makes it a valid biomarker for targeted binding to CD44 expressing cells. The observed molecular weights of the proteins were in accordance with the predicted for CD44 and β-actin.

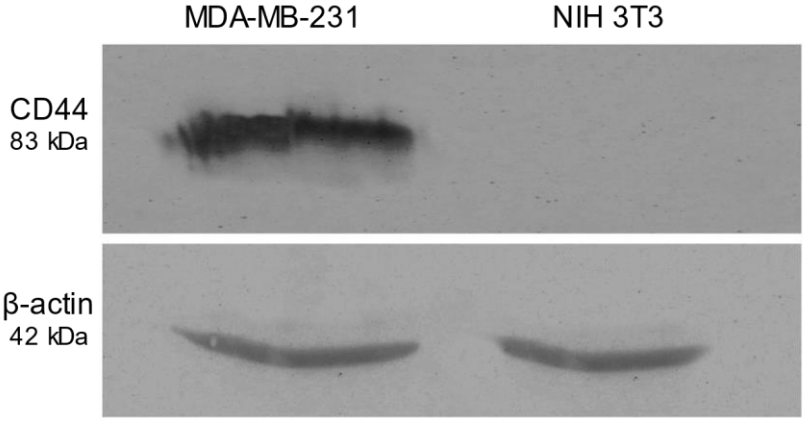

Figure S3. Western blot analysis of CD44 expression in two different cell lines, showing that the CD44 biomarker is expressed in MDA-MB-231 cells but not in NIH 3T3 cells. β-actin is used for protein normalization.

4.3. Cell Viability Assay

The cytotoxicity of the anti-CD44 mAb-QD NAs was measured with the colorimetric MTT (3-(4,5-dimethylthazolk-2-yl)-2,5-diphenyl tetrazolium bromide) assay. $1x10^5$ MDA-MB-231 cells were seeded in 96-well plates and cultured for 24 h at 37 °C and 5 % CO_2. Owing to the fact that the mAb-QD NAs were initially dispersed in DMSO:PBS (5:95) at a concentration of 0.5 µM (expressed as QD concentration), a minimum 10-fold dilution was required to have finally a concentration of 0.5% DMSO, which is compatible with cells. This means that the maximum concentration of QD NAs for avoiding toxicity due to DMSO is fixed at 50 nM. Cells were incubated with mAb-QD NAs at different concentrations, ranging from 5 nM to 50 nM (expressed in terms of QD concentration) for different times (3 h, 6 h, and 12 h) at 37 ˚C and 5% CO_2. Cells incubated with medium containing 0.5% DMSO were used as control. After incubation, cells were washed twice with 100 µL of cell medium and incubated for 4 h at 37 °C and 5% CO_2 with 100 µL of medium containing MTT at the dilution of 1:20. Afterwards, the medium was removed, 150 µL of DMSO was added to each well, and the solution was vigorously mixed to dissolve the produced formazan crystals as a result of the dehydrogenase mitochondrial activity of

living cells. The absorbance of each well was measured at 570 nm in a BioTek microplate reader. The cell viability V was calculated as a percentage of absorbance of treated cells related to the untreated control cells and is represented as mean of triplicates ± standard deviation (SD); Figure S4 [6].

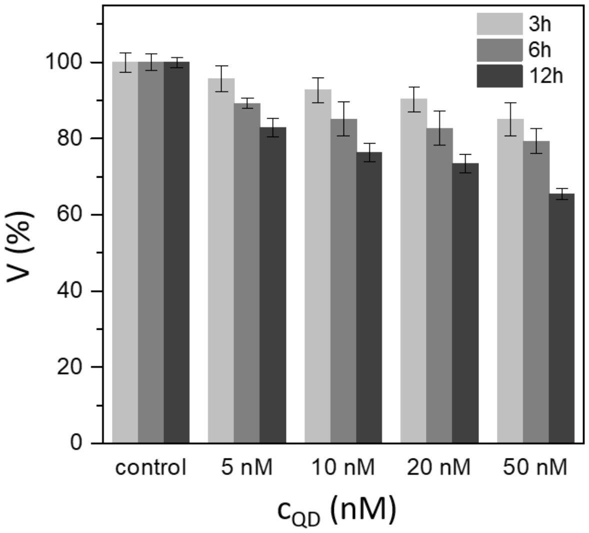

Figure S4. Cell viability of MDA-MB-231 cells after 3, 6, and 12 h of incubation with mAb-QD NAs at different concentrations (5 nM – 50 nM) as determined by MTT assay. Results are expressed as mean percentage ± SD from three independent experiments.

4.4. Imaging

Cells were imaged employing a confocal laser scanning microscope (CLSM 510 Meta) from Zeiss and using a Plan-Apochromat 63x/1.4 M27 oil immersion objective. mAb-QD NAs internalized by cells were excited with a 405 nm laser diode, and their fluorescence emission was collected using a LP 560 emission filter. Transmission images were also collected. Images were generated with the Zeiss AxioVision software, and were treated with ImageJ.

Uptake of anti-CD44 mAb-QD NAs by MDA-MB-231 ($CD44^+$) and NIH 3T3 ($CD44^-$) cell lines was investigated by CLSM. Cells were seeded in 8-well Lab-TekR Chambered Coverglass (Nunc, USA) at a density of $1x10^5$ cells per well, and were cultured overnight at 37 °C and 5% CO_2. The next day, the medium was removed and replaced by fresh medium containing non-modified QD NAs and anti-CD44 mAb-QD NAs at a concentration of 10 nM (expressed as QD concentration), and cells were incubated for 6 h at 37 °C for allowing the internalization of the particles. After incubation, cells were washed twice with 100 µL of cell medium to remove non-internalized or unbound particles, cells were then fixed using 4% paraformaldehyde (PFA) for 20 min at room temperature, and were washed extensively with PBS. For microscopy fixed cells supplemented with fresh PBS (500 µL) were imaged, and all confocal fluorescence images were acquired using the same imaging settings.

An example is shown in Figure S5 for the uptake of anti-CD44 mAb-QD NAs MDA-MB-231 ($CD44^+$) and NIH 3T3 ($CD44^-$) cells. Note that the results for MDA-MB-231 ($CD44^+$) and NIH 3T3 ($CD44^-$) cannot be directly compared, as in general cells have different uptake kinetics also in the case of nonspecific uptake, and thus differences here cannot be attributed to the presence of absence of CD44.

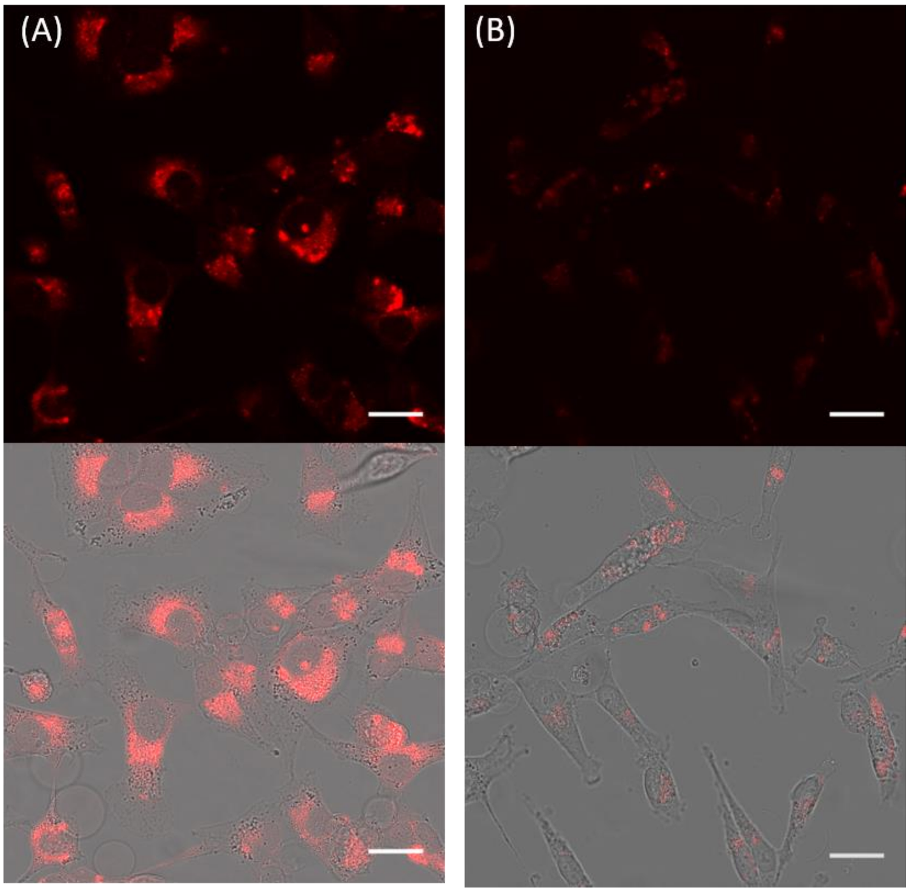

Figure S5. Representative confocal microscopy images of cells incubated with mAb-QD NAs (10 nM, 6 h, 37 °C): (**A**) MDA-MB-231 ($CD44^+$) cells treated with mAb-QD NAs; (**B**) NIH 3T3 ($CD44^-$) cells treated with mAb-QD NAs. Fluorescence was collected using a LP 560 emission filter under excitation at 405 nm and using a 63x objective. In the upper line the fluorescence channel with the fluorescence of the QD NAs displayed in red is shown, and is merge with the transmission channel as shown in the lower line. The scale bars correspond to 25 μm.

4.5. Flow Cytometry

Flow cytometry (FC) analysis was performed using a BD LSR IITM cytometer (BD Biosciences) equipped with an excitation laser 405 nm and a 605 ± 12 nm bandpass filter. The fluorescence signal was read using the FL3 channel. The mean fluorescent intensity (MFI) I_{MFI} was calculated from the histograms using the software FlowJo (version 7.6.1). Only population of viable cells was gated and used for the analysis of the cellular fluorescence intensity. Fluorescence of at least 10^4 cells per sample was analyzed and autofluorescence of cells was estimated using free mAb-treated cells as a control. Statistical data analysis was performed using unpaired Student's t-tests with $p< 0.05$ as the minimal level of significance.

FC analysis was performed for further analyzing the uptake of anti-CD44 mAb-QD NAs QDs. To this end, cells were seeded on 48-well plates and cultured for 24 h at 37 °C and 5% CO_2 until they reached 80% confluency. Then cells were incubated with fresh medium containing anti-CD44 mAb-QD NAs at a concentration of 10 nM (expressed as QD concentration), and cells were incubated at 37 °C for allowing the internalization of the particles. At different time points (1 h, 3 h, and 6 h) the solution was removed and cells were washed three times with cold PBS buffer. Cells were trypsinized, transferred into Eppendorf tubes, and collected by low-speed centrifugation (120 rcf, 5 min). After two washing steps, the pellet was resuspended in PBS (300 μL), and cells were analyzed by FC. An example is shown in Figure S6.

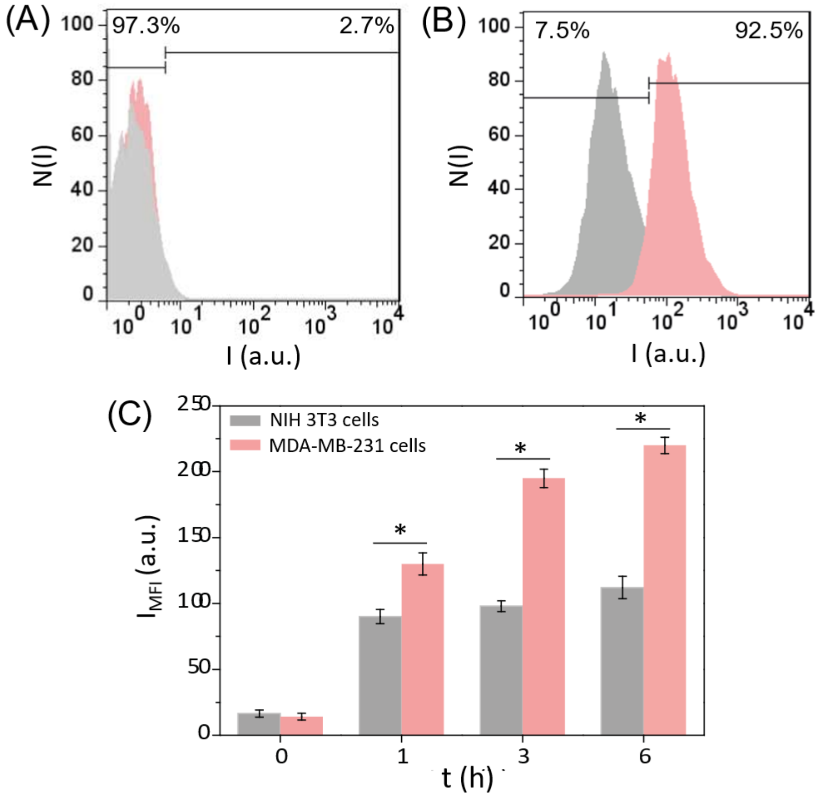

Figure S6. Flow cytometry (FC) analysis of the uptake of mAb-QD NAs by MDA-MB-231 cells (pink profile) and NIH 3T3 cells (gray profile). (**A**) FC histogram showing the distribution N(I) of the detected fluorescence I per cell of both cell lines before exposure to mAb-QD NAs (at time 0), showing that the autofluorescence from cells is negligible in the collected fluorescent channel. (**B**) FC histogram of both cell lines after treatment with mAb-QD NAs (10 nM) for 6 h. (**C**) Time course of cellular uptake from 0 h to 6 h, given as mean fluorescence intensity I_{MFI}. *represents $p < 0.05$ after unpaired Student's t-test.

References

1. Hühn, J.; Carrillo-Carrion, C.; Soliman, M. G.; Pfeiffer, C.; Valdeperez, D.; Massod, A.; Chakraborty, I.; Zhu, L.; Gallego, M.; Yue, Z.; Carril, M.; Feliu, N.; Escudero, A.; Alkilany, A. M.; Pelaz, B.; del Pino, P.; Parak, W. J. Selected Standard Protocols for the Synthesis, Phase Transfer, and Characterization of Inorganic Colloidal Nanoparticles. *Chem. Mater.*, **2017**, *29*, 399–461.
2. Michelena, O.; Padro, D.; Carrillo-Carrión, C.; del Pino, P.; Blanco, J.; Arnaiz, B.; Parak, W. J.; Carril, M. Novel fluorinated ligands for gold nanoparticle labelling with applications in 19F-MRI. *Chem. Comm.*, **2017**, *53*, 2447–2450.
3. Gallo, J.; García, I.; Padro, D.; Arnáiz, B.; Penadés, S. Water-soluble magnetic glyconanoparticles based on metal-doped ferrites coated with gold: Synthesis and characterization. *J. Mater. Chem.*, **2010**, *20*, 10010-10020.
4. Carrillo-Carrion, C.; Atabakhshi-Kashi, M.; Carril, M.; Khajeh, K.; Parak, W. J. Taking Advantage of Hydrophobic Fluorine Interactions for Self-Assembled Quantum Dots as a Delivery Platform for Enzymes. *Angew. Chem. Int. Ed.*, **2018**, *57*, 5033–5036.
5. Johnton, A. P. R.; Kamphuis, M. M. J.; Such, G. K.; Scott, A. M.; Nice, E. C.; Heath, J. K.; Caruso, F. Targeting Cancer Cells: Controlling the Binding and Internalization of Antibody-Functionalized Capsules. *ACS Nano,* **2012**, *6*, 6667–6674.
6. Ma, X.; Hartmann, R.; Jimenez de Aberasturi, D.; Yang, F.; Soenen, S. J. H.; Manshian, B. B.; Franz, J.; Valdeperez, D.; Pelaz, B.; Feliu, N.; Hampp, N.; Riethmüller, C.; Vieker, H.; Freese, N.; Gölzhäuser, A.; Simonich, M.; Tanguay, R.; Liang, X.-J.; Parak, W. J. Colloidal Gold Nanoparticles Induce Changes in Cellular and Subcellular Morphology. *ACS nano*, **2017**, *11*, 7807–7820.
